# Body mass index is an overlooked confounding factor in existing clustering studies of 3D facial scans of children with autism spectrum disorder

**DOI:** 10.1038/s41598-024-60376-0

**Published:** 2024-04-30

**Authors:** Martin Schwarz, Jan Geryk, Markéta Havlovicová, Michaela Mihulová, Marek Turnovec, Lukáš Ryba, Júlia Martinková, Milan Macek, Richard Palmer, Karolína Kočandrlová, Jana Velemínská, Veronika Moslerová

**Affiliations:** 1 Department of Biology and Medical Genetics, 2nd Faculty of Medicine, Charles University in Prague and Motol University Hospital, Prague, Czech Republic; 2 PRENET - Laboratoře Lékařské Genetiky s.r.o., Pardubice, Czech Republic; 3 Faculty of Science and Engineering, Curtin University, Perth, Australia; 4 Department of Anthropology and Human Genetics, Faculty of Science, Charles University, Prague, Czech Republic

**Keywords:** Autism spectrum disorders, 3D morphometry, Cluster analysis, Facial landmarks, 3-D reconstruction, Human behaviour, Molecular medicine

## Abstract

Cluster analyzes of facial models of autistic patients aim to clarify whether it is possible to diagnose autism on the basis of facial features and further to stratify the autism spectrum disorder. We performed a cluster analysis of sets of 3D scans of ASD patients (116) and controls (157) using Euclidean and geodesic distances in order to recapitulate the published results on the Czech population. In the presented work, we show that the major factor determining the clustering structure and consequently also the correlation of resulting clusters with autism severity degree is body mass index corrected for age (BMIFA). After removing the BMIFA effect from the data in two independent ways, both the cluster structure and autism severity correlations disappeared. Despite the fact that the influence of body mass index (BMI) on facial dimensions was studied many times, this is the first time to our knowledge when BMI was incorporated into the faces clustering study and it thereby casts doubt on previous results. We also performed correlation analysis which showed that the only correction used in the existing clustering studies—dividing the facial distance by the average value within the face—is not eliminating correlation between facial distances and BMIFA within the facial cohort.

## Introduction

Autism spectrum disorders (ASD) represent a common group of neurodevelopmental disorders. Their incidence is estimated at 1:59 individuals, and the internationally declared prevalence in medical literature is 1–2%. Males are four times more likely to be affected^1^. Complex interactions of genetic, environmental, and epigenetic factors, including the influence of genetic imprinting, are responsible for the development of ASD^2^.

ASD is either a non-syndromic (idiopathic ASD) or a concurrent symptom of a specific genetic syndrome (secondary ASD)^3^. Inheritance of ASD ranges between monogenic and multifactorial and is influenced by other factors that are thus far unknown or unquantifiable^4^. The clinical presentation of ASD is highly variable and includes three core symptoms: impairment of verbal and nonverbal communication, impaired social interactions, and stereotypical behaviors^5^.

The number of confirmed or suspected ASD-related genes is estimated in the hundreds to thousands range^6^. In some patients, an unambiguous genetic etiology can be demonstrated by detecting a single genetic variant. The genetic architecture of ASD, however, is more complicated and comprises single nucleotide variants (SNV) but also copy number variants (CNV), together with their incomplete penetrance, and variable expression^7^. Some studies suggest a “multi-hit” model for ASD, i.e., two or more “weaker” variants with less impact acting in synergy^8,9^.

Severe forms of ASD could be diagnosed as early as 18 months of age, while its milder forms are usually recognized in the preschool period^10^. Identification of ASD subgroups using endophenotype is rather difficult considering the substantial intrafamilial and interfamilial variability of ASD behavioural phenotypes^2^. Previous attempts at patient stratification (henceforward “clustering”) were based mostly on cognitive/psychomotor functions^5,11–15^. Some authors tried to cluster patients using electroencephalography (EEG) or brain magnetic resonance imaging (MRI)^16,17^. The two-dimensional analysis of patients´ photographs provided evidence that there is no characteristic “facies” which would allow reliable clinical/visual diagnosis of ASD. Nonetheless, several facial features that are more commonly found in patients with ASD have been described. These include a broader upper face, shorter mid-face, wider eyes, larger mouth, and philtrum^18^.

The use of three-dimensional (3D) geometric facial morphometry as an additional tool for the stratification of ASD patients was first reported by Aldridge et al.^19^. The authors analyzed 3D facial scans of 65 ASD boys (8–12 years of age). A total of 136 facial distances between defined anthropometric landmarks of each scan were analyzed. Most formed a cluster that overlapped with controls. However, there were two smaller clusters outside the main group representing both “extremes of the ASD clinical spectrum”- one comprised patients with a significantly worse clinical presentation of ASD, while the other had patients with mostly milder symptoms of Asperger’s syndrome.

Subsequently, Obafemi-Ajayi et al.^20^ ran a more detailed analysis on 62 ASD boys of the same age range whilst 83% of patients were from the same cohort used by Aldridge et al. The team used geodesic distances between defined facial landmarks. Only ASD patients entered clustering and subsequent statistical analyses. Authors showed that three clusters resulting from cluster analysis differ significantly in several cognitive measures related to ASD severity. One cluster exhibited both the higher separation from others in terms of facial distances and also worse clinical presentations of ASD. Furthermore, two other clusters had a significantly higher representation of Asperger’s syndrome patients and milder ASD severity. The controls were used only in PCA analysis where they overlapped with two clusters with milder ASD severity on the PCA plot but not with the third cluster with worst ASD cases.

Here we describe the relationship between the severity of ASD and 3D facial measurements in Czech pediatric patients of both sexes. We elucidate the central role of obesity on the cluster structure and consequently on its association with autism severity. This factor was not taken into account in the above mentioned studies.

## Methods

### Patient cohort

All patients were clinically diagnosed with ASD according to consensus criteria^21^. The lower age limit was eight years and the upper age limit was < 12 years, with cases ≥ 12 years being excluded from this study. This age limit was chosen so that patient skulls, which are the basis for facial dimensions, have finished the majority of their growth but have not yet started to change with the onset of puberty.

The following exclusion criteria were applied (number of excluded scans in parenthesis):

1. Patients with a clear laboratory genetic diagnosis of another underlying genetic disorder, i.e. syndromic ASD patients (31 scans)
2. Patients with other clearly defined environmental causes, e.g., fetal alcohol syndrome, fetal valproate syndrome (1 scan)
3. Patients of other than European—Caucasian origin (1 scan)
4. Patients born before the 35th week of gestation (8 scans)
5. Patients with poor quality 3D scans (14 scans)

Altogether, 92 male and 24 female non-syndromic ASD patients with high-quality 3D scans were analyzed. Out of these, seven male and three female patients had their mouths open during the 3D scan. Another three of the included male scans had severe obesity (body mass index for age (BMIFA) >  + 2SD). Scans of 106 healthy males and 51 healthy females of the same age range were used as controls. Controls were scanned using the same hardware (see further) and were provided by the Laboratory for 3D Imaging and Analytical Methods, Department of Anthropology and Human Genetics, Faculty of Science, Charles University.

ASD severity score was assessed clinically in all patients based on the extent of intellectual disability, functioning, and other behavioral symptoms^21^. The severity was assessed as mild, moderate, or severe (Table 1).

**Table 1:** Assessment of patients ASD symptoms´ severity in percentages.

	Mild % (#)	Moderate % (#)	Severe % (#)
Boys closed mouth	57.6% (49)	31.8% (27)	10.6% (9)
Girls closed mouth	57.1% (12)	38.1% (8)	4.8% (1)
Boys open mouth	14.28% (1)	42.86% (3)	42.86% (3)
Girls open mouth	33.3% (1)	66.7% (2)	0% (0)

Absolute numbers in brackets.

### 3D facial gestalt scanning

**Figure 1:**
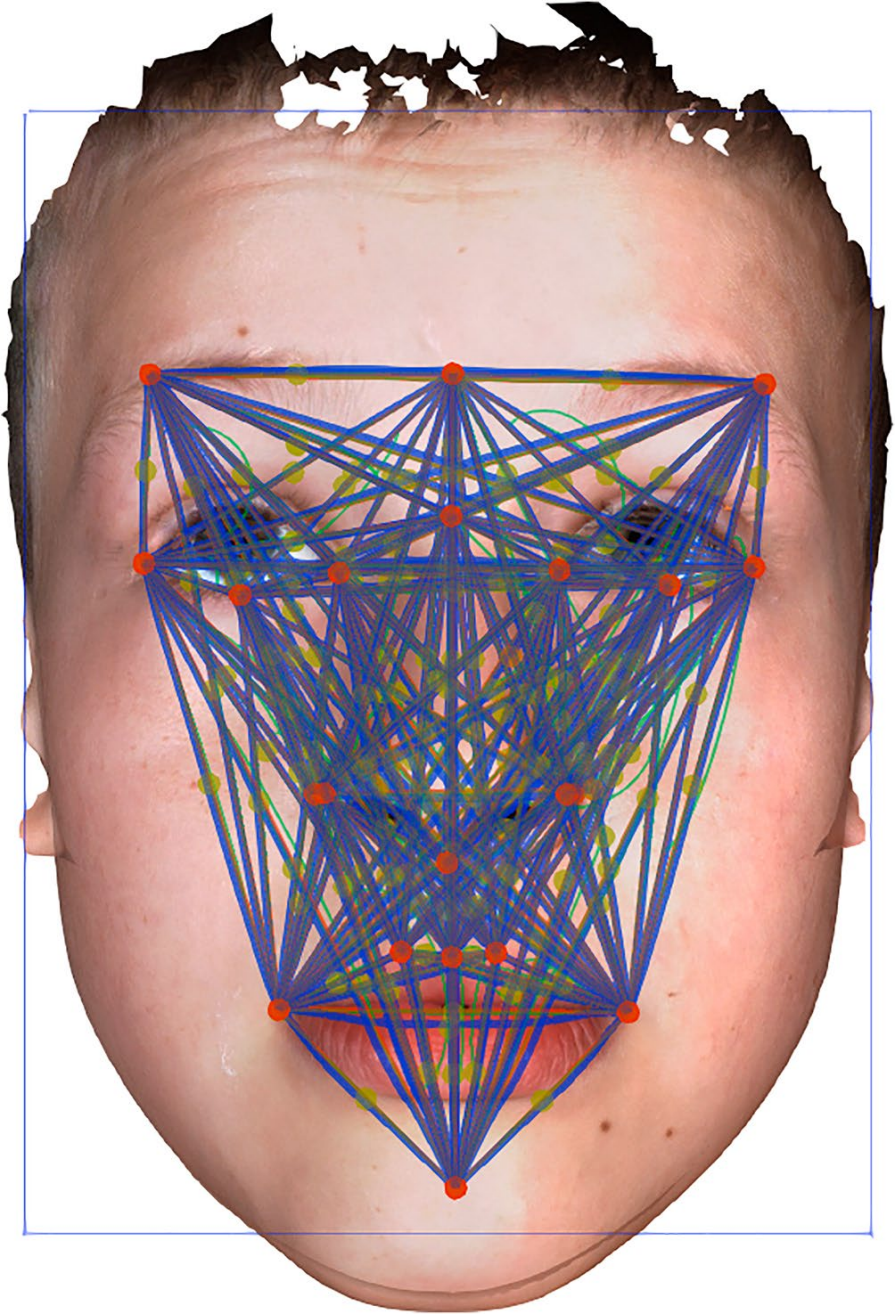
Measured distances (blue) between the 19 landmarks used (red). Landmarks comprised: glabella, nasion, subnasale, labrale superius, pogonion, superciliare lateralis right, superciliare lateralis left, exocanthion right, exocanthion left, palpebrale inferius right, palpebrale inferius left, endocanthion right, endocanthion left, alare right, alare left, cheilion right, cheilion left, crista philtri right, crista philtri left. Green dots—unused landmarks.

Non-invasive optical 3D scanning was carried out using the multi-camera non-contact 3dMDface System scanner (3dMD Ltd., London, United Kingdom; www.3Dmd.com). Each patient was scanned from a frontal view, with the head in a natural position and was instructed to have a neutral facial expression. Age-appropriate cartoons were played on a TV in front of the children to attract their attention and “stabilize” their frontal view towards the 3D camera. Scans were edited using the Cliniface (www.cliniface.org^22^ or Geomagic Wrap 2017 analytical software (www.3dsystems.com^23^. Landmarks were placed automatically by Cliniface, representing defined consensus anthropological points of the face^24^. Nineteen landmarks were used for the analysis^20^. Positions of the nineteen landmarks were verified manually in each studied case (Fig. 1).

### Data analysis

The .csv files with coordinates of the 19 landmarks were imported to R^25^, and Euclidean distances for each pair of landmarks in each proband were calculated. The corresponding geodesic distances were calculated using the Fast-Marching Toolbox in the Matlab environment^26,27^. One hundred seventy-one Euclidean distances per proband and another set of 171 geodesic distances per proband were obtained.

The following procedure is identical for Euclidean and geodesic distances and was performed in R.

Normalization was performed for each proband separately—all distances associated with individual proband were divided by their mean.

BMI was normalized concerning age by computing z-scores using the following equation: $BMIFA=\frac{BMI-{\mu }_{age}}{{sd}_{age}},$ where BMIFA is the age-normalized BMI used for all computations in this work, BMI in the equation corresponds to the BMI value of individual probands, μ_age_ denotes the average BMI value within the age group to which the proband belonged, and sd_age_ is the standard deviation within the same age group. (By the age group we mean tabularized age intervals in which we assume a constant BMI).

#### Correlation analysis

The Spearman correlation coefficient was calculated between the BMIFA and the distance associated with the above-mentioned landmark pairs for each proband group and each landmark pair^28^. The Spearman coefficient was used because the data are not normally distributed. Separated distributions of correlation coefficients for each proband group were obtained this way. Replacing BMIFA with age, analogical distributions were also computed for age; this allowed us to study how landmark distances are affected by age and BMIFA and how this relationship was affected by distance normalization.

#### Hierarchical clustering procedure

A hierarchical clustering procedure was used to elucidate the cluster structure for the proband groups. Every proband is represented by a vector of distances between all 19 landmarks used in this study and thus has a length of 179. Manhattan distances between all probands/vectors were then computed as an input to hierarchical clustering analysis. Final clustering was performed using the R function—hclust^25^, with the agglomerative method parameter set to the average.

The Calinski-Harabasz index was computed for every partition generated by the clustering procedure to select the technically best partition to divide into clusters^29^. The partition with the largest Calinski-Harabasz index was used for subsequent analyses.

We performed multiple instances of cluster analysis with different cohorts. We defined three clustering scenarios (s1, s2, s3) which we applied separately to the male and female patient groups and the linear and geodesic distances. We, therefore, ran the cluster analysis 12 times in total.

s1: In the first clustering scenario, all ASD probands were used together with all controls. Furthermore all combinatorically possible distances within the set of landmarks on each face^20^ were used in the clustering analysis.
s2: The second scenario was defined as s1, in which we filtered out some probands and distances. Specifically, we removed subjects with open mouths and probands identified as outliers using Rosner’s outlier test^30^. We then removed all inter-landmark distances from the analysis exhibiting a significant correlation with BMIFA after Bonferroni correction^31^ to reduce the effect of confounding factors.
s3: The third clustering scenario differs from s2 only in the distances used for analysis. As in s2, subjects with open mouths and outliers were removed; however, in s3, we only used distances our anthropologist chose that were not expected to correlate with BMIFA (listed in Supplemental Table 1). Distances were chosen to be minimally influenced by BMIFA.

#### Statistical testing

Kruskal Wallis test was used to determine if significant differences between mean BMIFA and age existed between clusters^32,^. If these tests were significant, the Mann–Whitney test was used to find all significant pairwise differences^33,34^. Resulting pairwise p values were corrected by Bonferroni correction in cases when the clustering analysis produced > 2 non-trivial clusters. A hypergeometric test was used to test the significance of the enrichment of individual phenotypic categories within clusters and also for enrichment of autism severity classes within the open-mouth proband group^35^. *P* values < 0.05 are considered statistically significant and all statistical testing was performed in R.

### Ethical approval and consent to participate

Fiduciaries of underaged patients underwent comprehensive genetic counseling followed by signing of informed consent, including approval of 3D facial scanning. The study was approved by the Internal Ethics Board of the Motol University Hospital and was carried out per the provisions of art. 28–29 of Act 373/2011 Coll, and in line with the World Medical Association the “Declaration of Helsinki” principles.

## Results

### Linear distances

**Figure 2:**
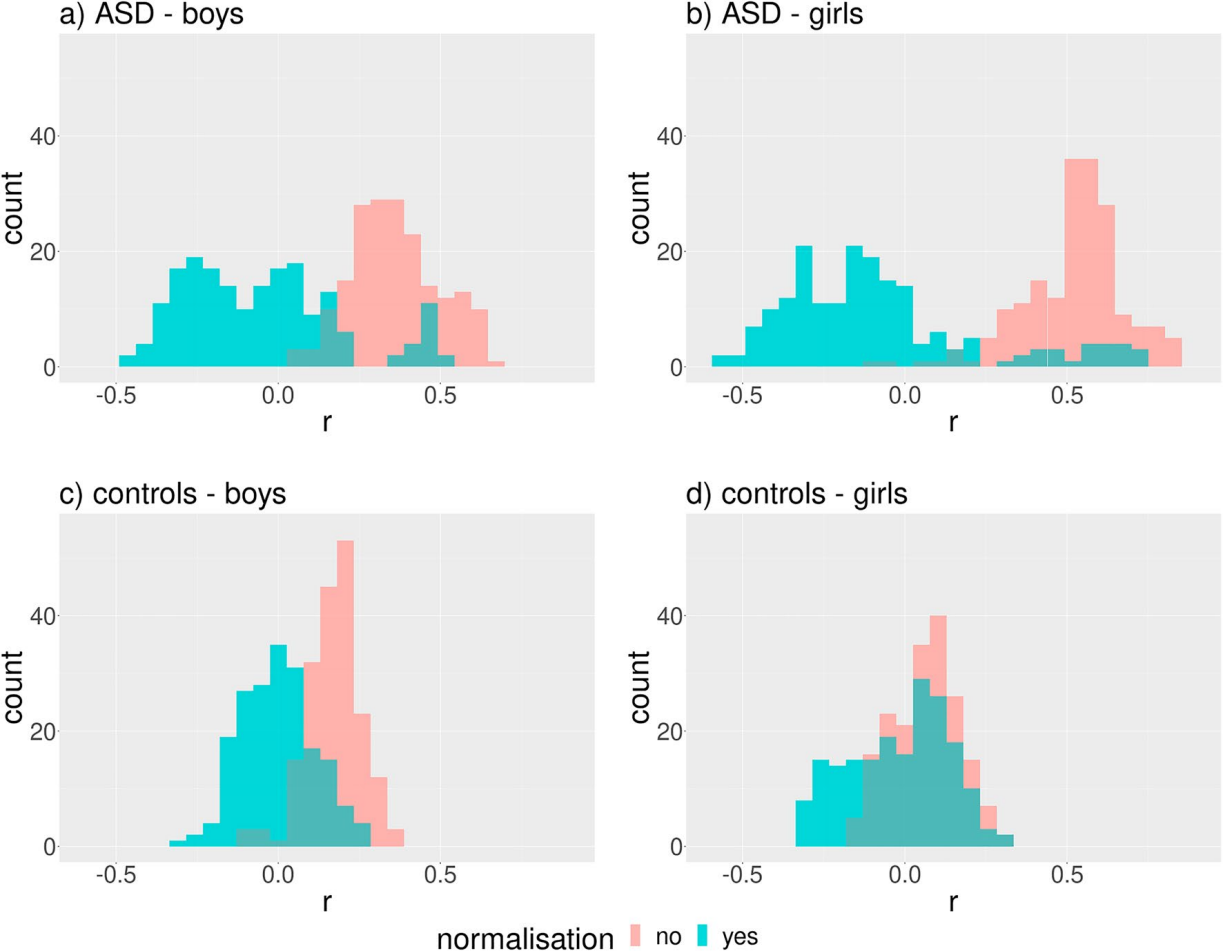
Distribution of Spearman’s correlation coefficient (r) between BMIFA and facial distances corresponding to different landmark pairs within the proband cohorts before and after normalization. The Euclidean norm was used for distance computations. Subparts: a) ASD male patients, b) ASD female patients, c) controls male, d) controls female.

Two preliminary correlation analyses took place in both sexes**.** First, we correlated BMIFA with all individual linear distances before and after their normalization. The distribution of Spearman’s coefficient was shifted to the right before normalization and yielded a majority of positive correlation coefficients (Fig. 2a–d). Interestingly, a high proportion of negative correlations appear after normalization (Fig. 2a–d), and a group of highly positive correlation coefficients remains on the right side of the distribution in ASD groups (Fig. 2a,b). These results demonstrated that normalization divided by the mean does not sufficiently eliminate correlations between BMIFA and landmark distances.

**Figure 3:**
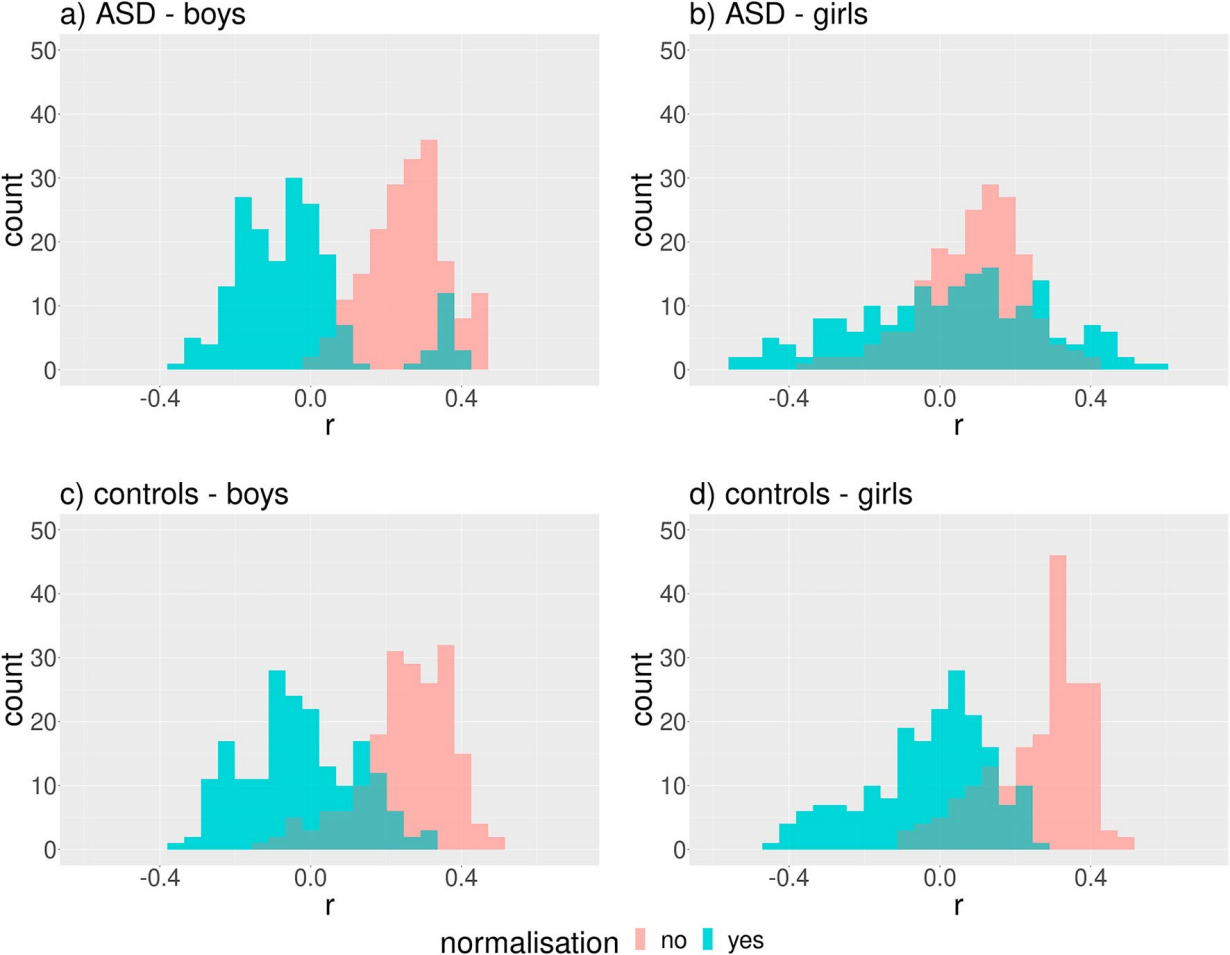
Distribution of Spearman’s correlation coefficient (r) between age and facial distances corresponding to different landmark pairs within the proband cohorts before and after normalization. The Euclidean norm was used for distance computations. Subparts: a) ASD male patients, b) ASD female patients, c) controls male, d) controls female.

We obtained very similar results for age (Fig. 3). Both findings indicate the possible influence of BMIFA and age on the clustering results.

#### Clustering analysis with all combinatorically possible distances between landmarks

**Figure 4:**
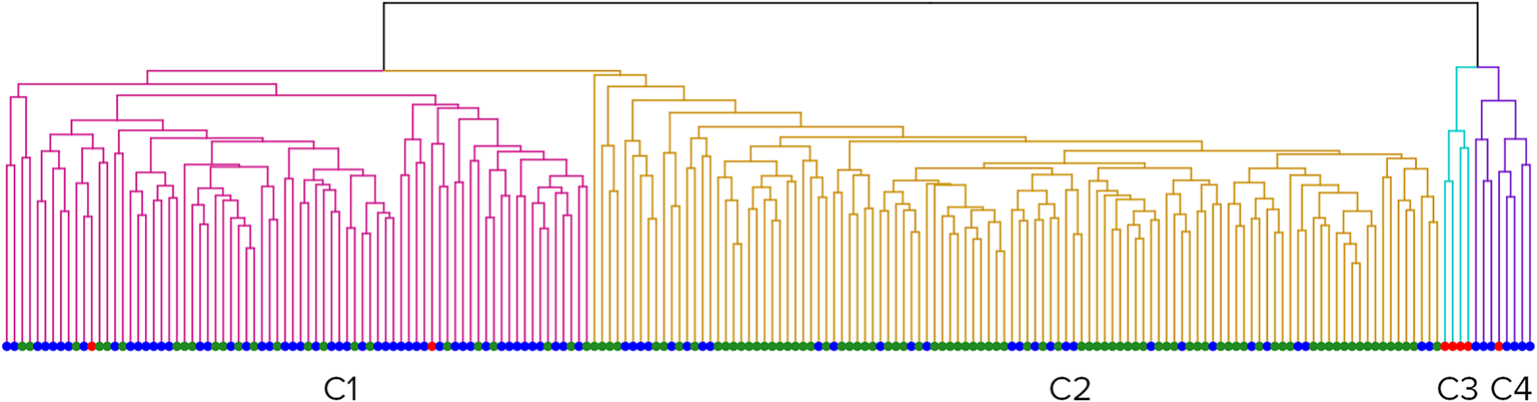
Clusters formed by all male scans. Red dots – open mouth patients, green dots – controls, and blue dots – ASD patients. From left to right: Cluster 1 (red), Cluster 2 (yellow), Cluster 3 (blue), Cluster 4 (purple).

Applying clustering scenario s1 on the male cohort with linear distances resulted in four clusters (Fig. 4).

The biggest cluster, Cluster 2, was significantly overrepresented with controls (74%, pval < 10^−10^). Cluster 1 (65%, pval < 10^−6^) and Cluster 4 (100%, pval = 0.012) were significantly overrepresented with ASD patients. The smallest cluster, Cluster 3, was composed exclusively of ASD patients with open mouths (100%, pval < 10^−6^).

Clusters exhibit significant differences in BMIFA (pval < 10^−7^), age (pval = 0.0002), and ASD severity (pval = 0.0021) according to the Kruskal Wallis test.

Cluster 1 showed significantly higher BMIFA (pval < 0.004) and age (pval < 0.004) than Cluster 2. Cluster 4 showed significantly higher BMIFA (pval < 0.004) and ASD severity (pval = 0.034) than Cluster 2.

In addition, the smallest cluster, i.e., 3, showed significantly higher ASD severity than cluster 1 and 2 (pval_3-1_ = 0.012, pval_3-2_ = 0.00228) but not BMIFA. All relevant cluster properties are summarized in Table 2.

**Table 2:** Male group. Characteristics of clusters.

Cluster designation	n	F (c)	pval	F (ASD)	pval	F (OM)	pval	mean(BMI)	mean(BMIFA)	sd(BMIFA)	mean(age)	sd(age)	# mild cases	# moderate cases	# severe cases
Cluster 1	76	0.31	0.99	0.65	2.7 × 10^−7^	0.02	0.82	19.1	0.59	1.39	10.6	1.65	27	19	6
Cluster 2	110	0.74	2.1 × 10^−11^	0.25	0.99	0	1	16.4	−0.19	0.7	9.7	1.25	21	5	2
Cluster 3	4	0	1	0	1	1	5.6 × 10^−7^	20.4	1.44	3.33	10	1.59	0	1	3
Cluster 4	8	0	1	0.87	0.01	0.12	0.25	21.1	1.48	1.41	10.3	1.46	2	5	1

Rows – Clusters 1, 2, 3, and 4. Columns – their respective properties: n – total cluster size; F (c) – the fraction of controls; pval—its significance; F (ASD) – the fraction of ASD patients, pval – its significance; F (OM) – the fraction of open mouth patients; Mean BMI; Mean BMIFA; sd(BMIFA)—standard deviation BMIFA; Mean age; sd(age)—standard deviation age; Number of mild cases; Number of moderate cases; Number of severe cases.

**Figure 5:**
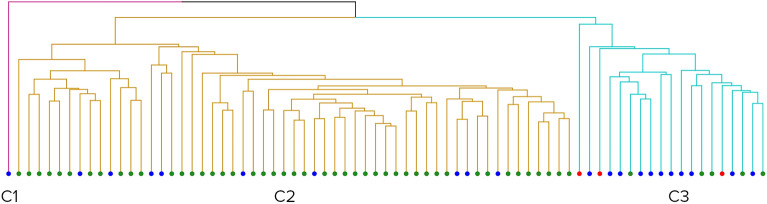
Clusters formed by all female scans. Red dots – open mouth patients, green dots – controls, and blue dots – ASD patients. From left to right: Cluster 1 (red), Cluster 2 (yellow), and Cluster 3 (blue).

Repeating the previous analysis (clustering scenario—s1) in the female group of patients resulted in three clusters, including one cluster (Cluster 1) with a single case (Fig. 5). Similarly to the previous case, the biggest cluster, Cluster 2, is significantly overrepresented with controls (84%, pval < 10^−5^), and Cluster 3 was significantly overrepresented with ASD patients (57%, pval = 0.001).

Cluster 3 showed significantly higher BMIFA (pval < 10^−4^) and age (pval = 0.023) than Cluster 2. There was no significant difference in ASD severity between Cluster 2 and 3. All relevant cluster properties are summarized in Table 3.

**Table 3:** Female group. Characteristics of clusters.

Cluster designation	n	F (c)	pval	F (ASD)	pval	F (OM)	pval	mean (BMI)	mean (BMIFA)	sd (BMIFA)	mean (age)	sd (age)	# mild cases	# moderate cases	# severe cases
Cluster 1	1	0	1	1	0.28	0	1	14.4	− 0.99	N/A	10.08	N/A	0	1	0
Cluster 2	55	0.83	4 × 10^−6^	0.16	0.99	0	1	16.1	− 0.18	0.95	10.25	1.19	5	4	0
Cluster 3	19	0.26	0.99	0.57	1.4 × 10^−3^	0.15	0.01	21	0.97	1.14	10.97	1.35	8	5	1

Rows – Clusters 1, 2, and 3. Columns – their respective properties: n – total cluster size; F (c) – the fraction of controls, pval—its significance; F (ASD) – the fraction of ASD patients, pval—its significance; F (OM) – the fraction of open mouth patients; Mean BMI; Mean BMIFA; sd(BMIFA)—standard deviation BMIFA; Mean age; sd(age)—standard deviation age; Number of mild cases; Number of moderate cases; Number of severe cases.

#### Clustering analysis with defined subsets of all distances

Applying clustering scenario s2 to the male cohort resulted in a partition with two clusters (of sizes 180 and 6). The two clusters do not show over-representation by any phenotypic category or significant differences in the examined phenotypic variables. Clustering of all females with the same clustering scenario resulted in 4 clusters, with one of the clusters having a size of one. Only one cluster was significantly overrepresented with ASD cases. In other words, the separation of controls from ASD cases was rather weak compared to scenario s1. The same cluster overrepresented with ASD cases had a significantly higher BMIFA than the remaining non-trivial clusters. No other significant differences between clusters were observed in scenario s2.

Applying clustering scenario s3 to the male cohort resulted in trivial partitions, where each proband corresponds to one cluster. Clustering of the female cohort with the same clustering scenario resulted in 3 clusters, with one cluster having a size of one. The two biggest clusters did not show significant over-representation by any of the examined phenotypic categories, and no significant difference in BMIFA or age existed between them. Interestingly, Cluster 2 showed significantly higher ASD severity than Cluster 1 (pval = 0.0226).

### Geodesic distances

**Figure 6:**
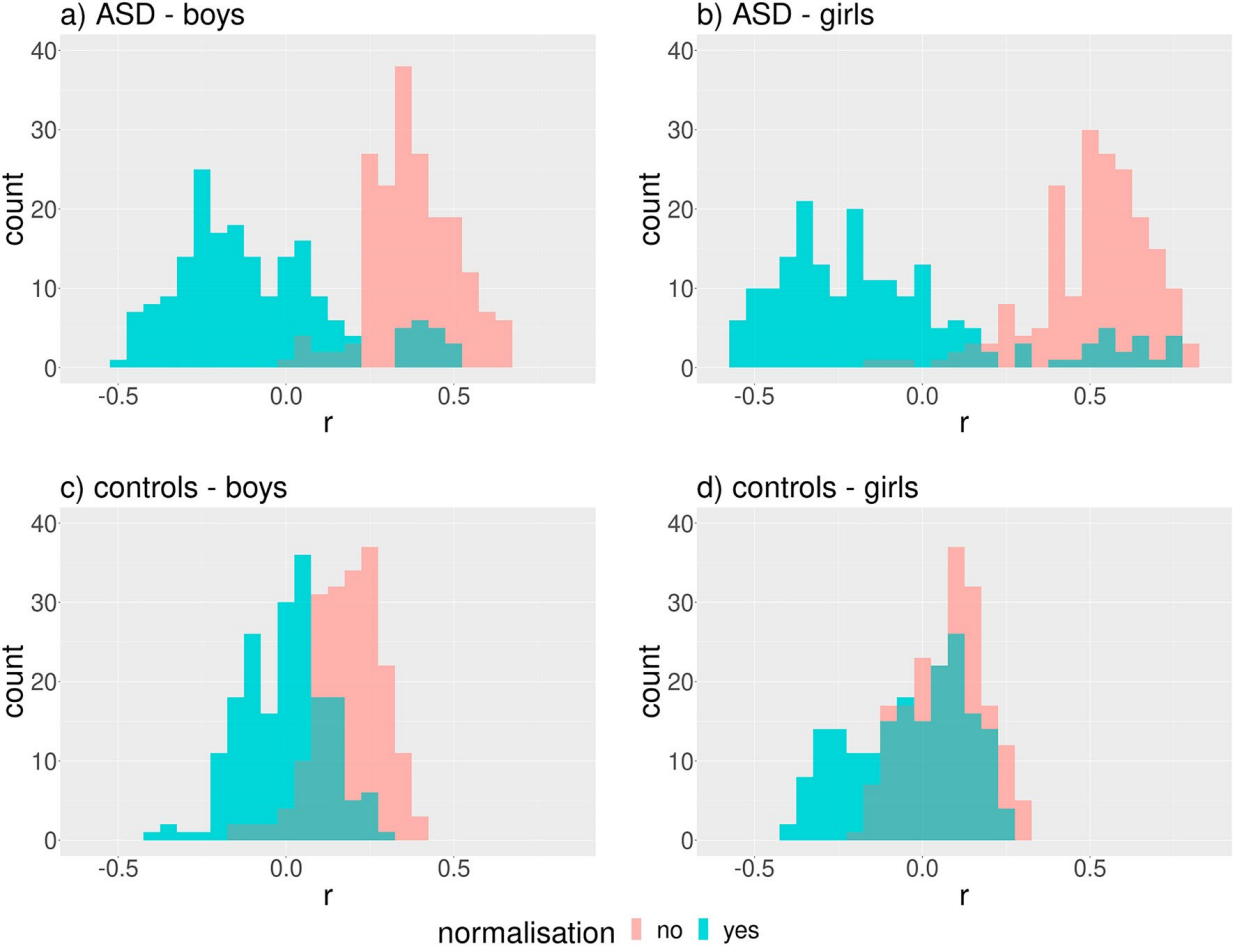
Distribution of Spearman’s correlation coefficient (r) between BMIFA and facial distances corresponding to different landmark pairs within the proband cohorts before and after normalization. The geodesic distances were used. Subparts: a) ASD male patients, b) ASD female patients, c) controls male, d) controls female.

**Figure 7:**
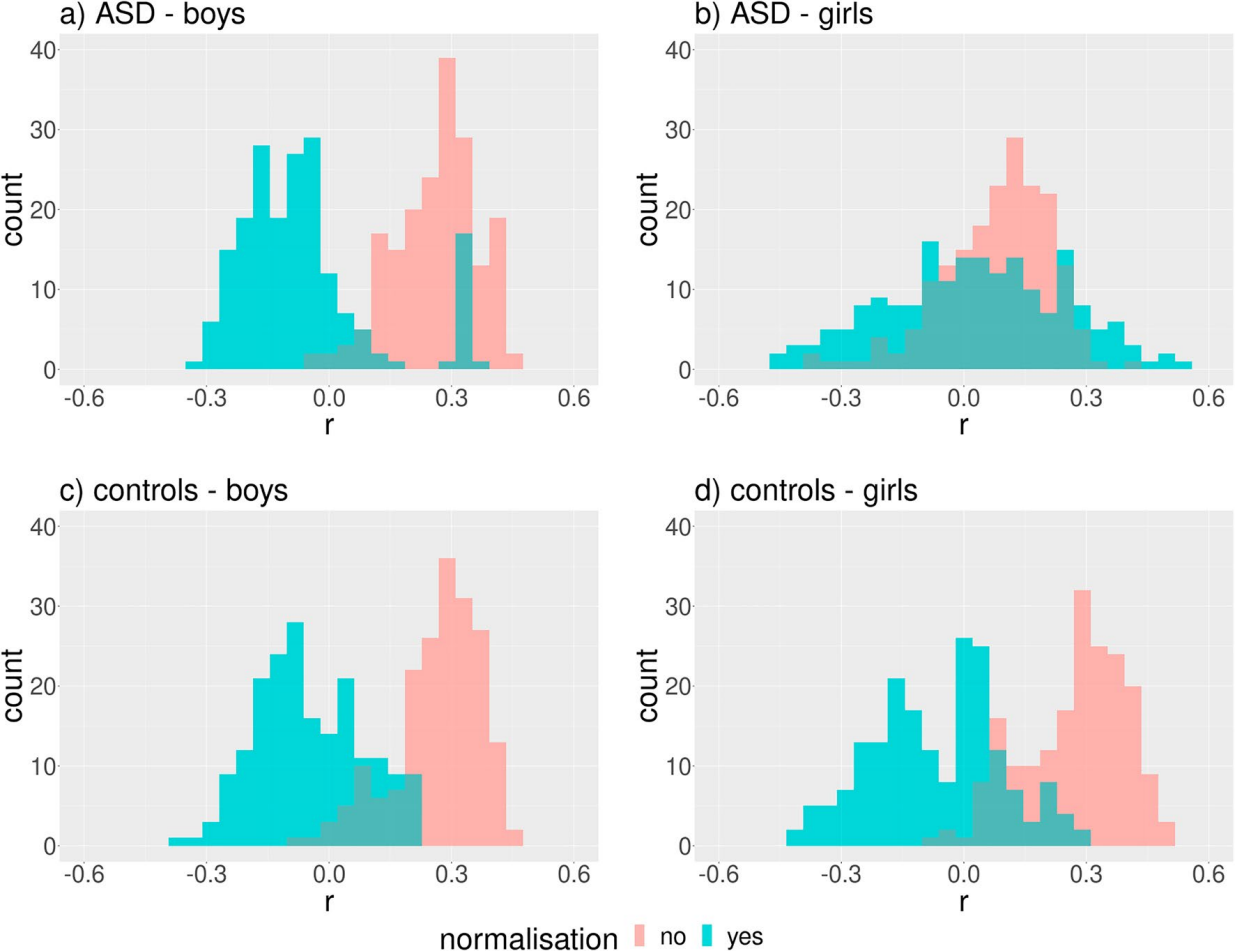
Distribution of Spearman’s correlation coefficient (r) between age and facial distances corresponding to different landmark pairs within the proband cohorts before and after normalization. The geodesic distances were used. Subparts: a) ASD male patients, b) ASD female patients, c) controls male, d) controls female.

Correlation analysis using geodesic distances resulted in qualitatively the same results as with linear distances (Figs. 6 and 7).

#### Clustering analysis with all combinatorically possible distances between landmarks

**Figure 8:**
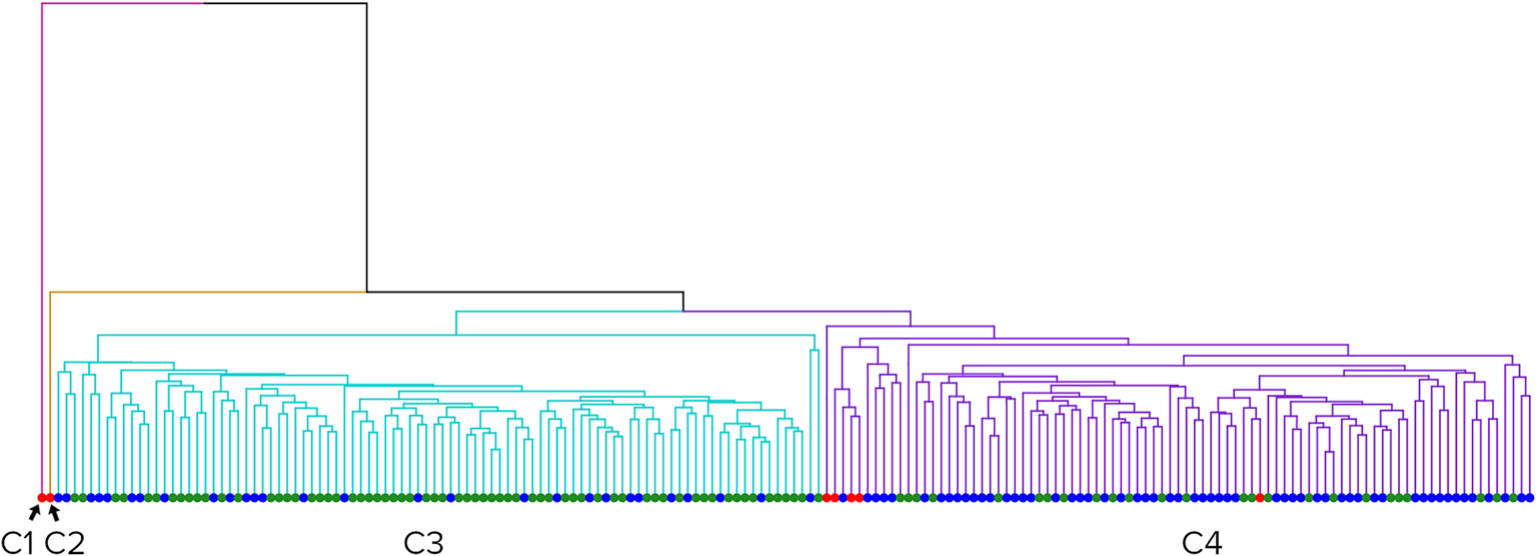
Clusters formed by all male scans when using geodesic distances. Red dots – open mouth patients, green dots – controls, and blue dots – ASD patients. From left to right: Cluster 1 (red), Cluster 2 (yellow), Cluster 3 (blue), and Cluster 4 (purple).

The resulting partition of clustering scenario s1 applied to the male cohort was composed of four clusters, with two clusters having a size of one (Fig. 8). The remaining two clusters were approximately the same size. Cluster 3 was significantly overrepresented with controls (70%, pval < 10^−8^), and Cluster 4 was significantly overrepresented with ASD patients (66%, pval < 10^−6^). Cluster 4 showed a significantly higher BMIFA (pval < 10^−7^), age (pval < 0.0008), and ASD severity (pval = 0.01437) than Cluster 3.

**Figure 9:**
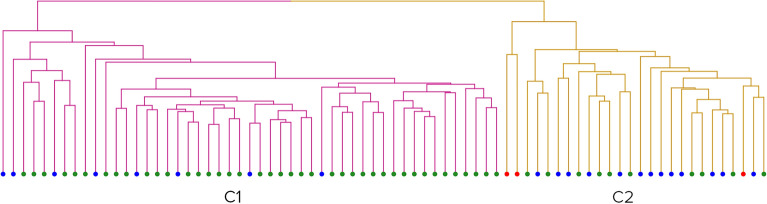
Clusters formed by all female scans when using geodesic distances. Red dots – open mouth patients, green dots – controls, and blue dots – ASD patients. From left to right: Cluster 1 (red) and Cluster 2 (yellow).

Repeating the previous scenario s1 for a female group of patients resulted in two clusters (Fig. 9).

Cluster 1 was significantly overrepresented with controls (84%, pval = 0.0001), and Cluster 2 was significantly overrepresented with ASD patients (50%). Cluster 2 showed significantly higher BMIFA (pval < 10^−4^) and age (pval = 0.017) than Cluster 1; however, this was not true for ASD severity.

#### Clustering analysis with subsets of all distances

Applying clustering scenario s2 to the male cohort resulted in three clusters (of sizes 160, 9, and 2 probands). Clusters do not show significant over-representation by any of the examined phenotypic categories nor significant differences between examined phenotypic variables. Repeating the same clustering scenario s2 with the female group resulted in three clusters (of sizes 52, 8, and 12). In contrast to the previous case, we observed significant differences in BMIFA between the resulting clusters. Cluster 3 showed a significantly higher BMIFA than Cluster 1 and 2 (pval = 0.012 and 0.003, respectively). No significant differences existed between clusters relative to the other examined phenotypic variables.

The clustering scenario s3 in both the male and female cohort resulted in trivial partitions, where every proband corresponded to a single cluster.

### Relationship with ASD severity

**Figure 10:**
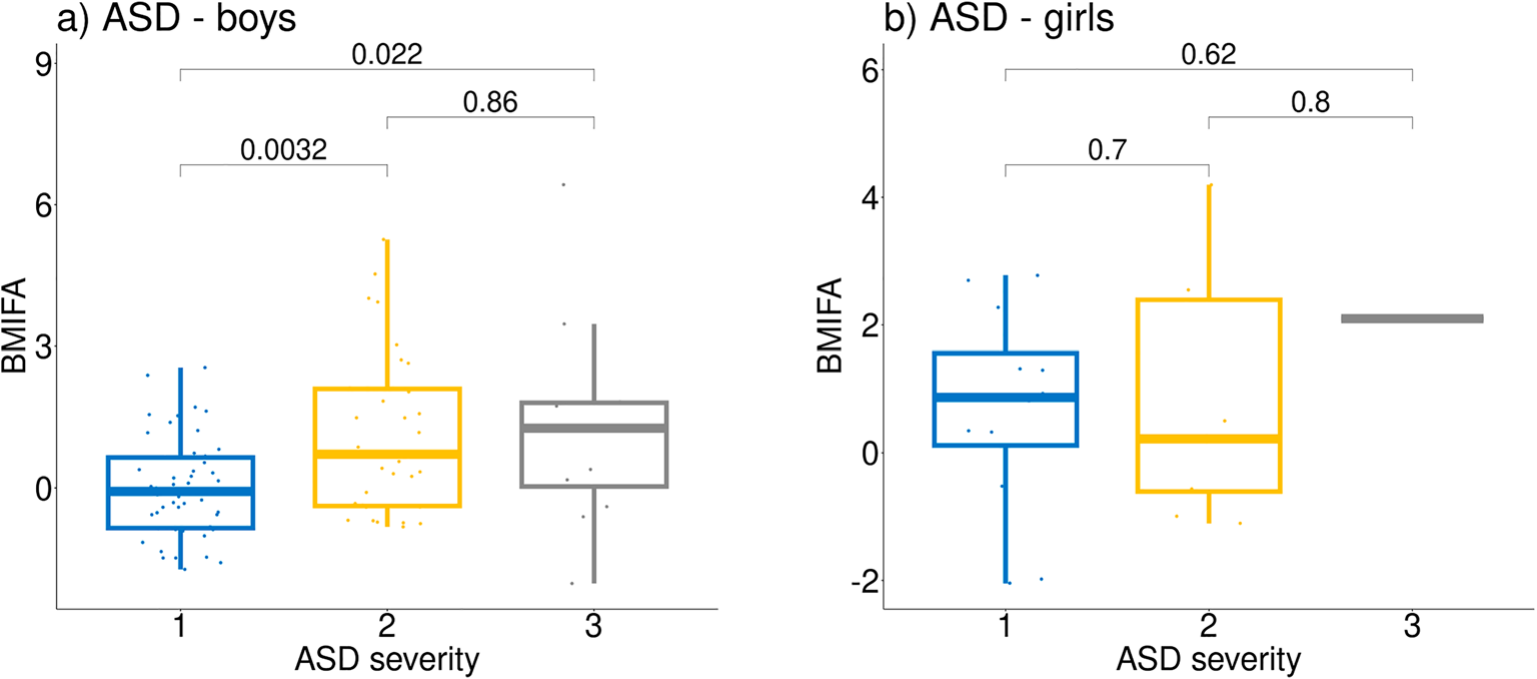
The relationship between ASD severity and BMIFA. Y-axis = BMIFA, X-axis = ASD severity (1 – mild, 2 – moderate, and 3 – severe). Subparts: a) ASD male patients, b) ASD female patients.

The above clustering results, specifically the association of clusters with both significantly different mean BMIFA and ASD severity, suggest that there could be a relationship between BMIFA and ASD severity. We found that ASD boys with higher values of ASD severity also had significantly higher BMIFA values implying a positive relationship between BMIFA and ASD severity (Fig. 10). In ASD girls, the relationship was not significant.

Correlating ASD severity with open or closed mouths demonstrated that the number of severe autism cases within the group of probands with open mouth is significantly higher than is expected by chance, pval = 0.005 (Table 1).

## Discussion

Our results within scenario s1 agreed with previous observations^20^ in that the clusters were associated with significantly different clinical courses of ASD in terms of its severity. However, beyond the results of previous studies^19,20^, we demonstrated that the observed clustering and consequently its association with ASD severity measure is determined by BMIFA, using both linear and geodesic distances.

This statement was confirmed by the scenario s2 analysis in which, after removing all outliers and all distances significantly correlating with BMIFA, the resulting clusters did not show an over-representation by any phenotypic category except single cluster in the case of female group and euclidean distances. We observed some residual differences between clusters in BMIFA, especially in case of geodesic distances, but this can be explained by imperfect elimination of BMIFA correlated distances when using Bonferroni correction. However, remarkable disappearance of ASD severity correlations and phenotypic category enrichment is obvious in this case. Using the inverted scenario (s3), where the distances were chosen to be as little influenced by BMIFA as possible, we got trivial partitions in all sub-scenarios except female cohort in case of euclidean distances This further suggests that apart from BMIFA, there were no other reasons for distance vectors/probands to cluster.

In the female group, the results were in partial agreement with the male group, i.e., we also observed significant separation between controls and ASD cases and differences in BMIFA and age, however, not in ASD severity. This may be explained by the smaller number of female scans available; thus, the statistical evidence for females was weaker. Therefore, a study using a larger number of female scans is needed to confirm our results.

The fact that removing BMIFA-related distances leads to the absence of clustering or produces clusters that are not correlated with clinical variables suggests that BMIFA strongly influences clustering results. Furthermore we showed that the commonly used normalization, i.e., division of facial distances by the mean value, does not eliminate correlations between individual facial distances and BMIFA. We also reproduced known results^36^, demonstrating a positive relationship between BMIFA and ASD severity (Fig. 10) in ASD boys. This fact and the BMIFA effect on clustering explain the correlation between clusters and clinical variables related to ASD severity observed in our work and previous works^19,20^.

In their retrospective study, Curtin et al.^37^ found that children with ASD were 40% more likely to be obese than children in the general population. Several factors may be associated with a higher risk of developing obesity in children with ASD, such as psychopharmacological treatments, sleep disorders, problems with engaging in sustained physical activity, and atypical eating habits^38^. Autistic children are notoriously picky eaters, often consuming only specific foods or foods with a certain consistency and presentation. It is not unusual for a child to “fear” trying new foods and eat relatively few food items^39^. Our work raises the question of whether all existing studies dealing with autistic faces clustering based on facial distances cannot be simplified to the equation “high BMIFA equals worse phenotype” because no BMIFA-based exclusion/correction processes are mentioned in the original publications^19,20^.

It is clear from our work that BMIFA should be considered in 3D facial soft tissue assessment since it can introduce a significant bias. In a study by Tan et al.^40^, BMIFA is considered, but the methodology is not entirely clear. Influence of BMI is examined by calculating facial areas between selected landmarks in ASD children. No significant differences were found. In 2020 Tan et al. examined non-autistic siblings of ASD children in a similar manner^18^, no significant differences were found. BMI was discussed marginally as a confounding factor in similar studies; it was not further considered.

We observed another confounding factor that can bias facial clustering studies, and to our knowledge, has never been mentioned before. We suspect that the authors of previous studies did not exclude 3D models of faces with open mouths. This is partially backed up by Fig. 5 in reference^20^, which includes a child with an open mouth. However, we were not able to establish contact with the authors for further clarification of this issue. Opening the mouth significantly increases the vertical distances of the face, and these patients could form their “own” cluster. On the other hand, an inability to persuade the child to close their mouth could mean the child does not have the necessary mental capacities or social skills to cooperate and thus might have a more severe behavioural phenotype. This could imply that the analysis correctly identified a group of patients with severe ASD phenotypes. However, this approach bears oversimplification by “automatically” associating an open mouth with a “worse” phenotype. This is not an observation that needs a 3D scanner-based validation. Therefore, in our opinion, these patients should be a priori excluded to get less biased results.

We assessed the severity of ASD clinical presentations from mild to severe (see Table 1) and observed that open-mouth patients have a higher percentage of moderate-to-severe phenotypes than closed-mouth patients, although it must be noted that the number of open-mouth patients available to our study was limited.

There are further potential limitations of our study. Although each patient was scanned from the frontal view with their head in a natural position and a neutral facial expression, there was one factor we could not eliminate, i.e., jaw clenching. A clenched jaw produces different vertical distances than a relaxed jaw, even though the mouth is closed. In our study, the children were shown cartoons to help them remain relaxed and not overly focused on the scanning process. This factor was not addressed in previous studies. Furthermore, the quality of 3D scans can vary according to the device and the degree of patient cooperation within the scanning process.

Another step in further objectifying the process and nullifying the influence of BMIFA is to remove the influence of soft tissues by analyzing landmarks on the cranial skeleton of probands. A brain MRI is a standard part of a clinical ASD workup. This approach was not used and is yet to be evaluated within the context of 3D facial scanning.

In summary, 3D facial scanning remains a promising non-invasive examination modality for objective assessment of patients´ facial phenotypes, whether for documenting purposes or automated human phenotype ontology (HPO) terms extraction. But it is very important that BMI is addressed in 3D scan facial studies to differentiate between traits based on skull features from those based on soft tissue changes. There are still many unknowns in ASD patient phenotypes, be it genetic, facial or behavioral. New methods could be valuable for ASD diagnostics, benefit patients and their families and aid the stratification of patients in terms of their severity and application of customized treatments.

### Supplementary Information

Supplementary Table 1.

## Data Availability

The datasets of patients used and/or analyzed during this study are available from the corresponding author upon reasonable request.
