# Supplementary material for: Body mass index is an overlooked confounding factor in existing clustering studies of 3D facial scans of children with autism spectrum disorder
Source: Sci Rep. 2024 Apr 30;14:9873. doi: 10.1038/s41598-024-60376-0 (PMC11059264; doi:10.1038/s41598-024-60376-0)
Supplement: Supplementary file 1 — Supplementary Table 1. [file 41598_2024_60376_MOESM1_ESM.docx]

**Supplemental table 1**

| **List of chosen distances** | **The key to the correspondences of used anthropometric landmarks with the used numeric abbreviation** |
| --- | --- |
| 6R-5R, 5R-5L, 42R-42L, 42R-8, 42R-6R, 4R-4L, 17R-17L, 17R-11, 4R-12, 4R-28, 17R-28, 1R-5R, 42R-2R, 42R-21R, 21R-22R, 21R-8, 6R-8, 22R-8, 6R-19, 5R-19, 22R-19, 22R-22L, 21R-22L, 1R-1L, 1R-8, 1R-19, 1R-24, 1R-12, 1R-28, 1R-23, 6R-2R, 6R-1R, 6R-24, 6R-28, 6R-23, 6R-8, 6R-19, 5R-2R, 5R-1R, 5R-24, 5R-28, 5R-23, 5R-8, 5R-19, 8-19, 8-24, 119-24, 8-31, 8-12, 8-11, 8-23, 19-24, 19-31, 19-12, 19-28, 19-11, 19-23, 24-31, 12-11, 12-28, 28-11, 28-23, 31-23, 31-12, 4R-4L, 2R-2L, 17R-17, 2R-12, 2R-11, 2R-23, 2R-31, 2R-24, 2R-8, 2R-19, 13-31, 1R-2R, 6L-5L, 42L-8, 42L-6L, 17L-11, 4L-28, 17L-28, 1L-5L, 42L-2L, 42L-21L, 21L-22L, 21L-8, 6L-8, 22L-8, 6L-19, 5L-19, 22L-19, 1L-8, 1L-19, 1L-24, 1L-12, 1L-28, 1L-23, 6L-2L, 6L-1L, 6L-24, 6L-28, 6L-23, 6L-8, 6L-19, 5L-2L, 5L-1L, 5L-24, 5L-28, 5L-23, 5L-8, 5L-19, 2L-12, 2L-11, 2L-23, 2L-31, 2L-24, 2L-8, 2L-19, 1L-2R | 8,"Glabella"  19,"Nasion"  31,"Subnasale"  12,"Labrale Superius"  23,"Pogonion"  42,"Superciliare Lateralis",R  42,"Superciliare Lateralis",L  6,"Exocanthion",R  6,"Exocanthion",L  22,"Palpebrale Inferius",L  22,"Palpebrale Inferius",R  5,"Endocanthion",R  5,"Endocanthion",L  1,"Alare",R  1,"Alare",L  2,"Cheilion",R  2,"Cheilion",L  4,"Crista Philtri",R  4,"Crista Philtri",L  17,"Mid-vermilion Inferius",L  17,"Mid-vermilion Inferius",R  11,"Labrale Inferius"  24,"Pronasale"  21,"Palpebrale Superius",R  21,"Palpebrale Superius",L  28,"Stomion Inferius" |

**Supplemental table 1** - Column one represents selected distances that should not correlate with BMI. Column two indicates what anthropometric landmarks correspond to which number, eg. 6R-5R means a distance between a right exocanthion and a right endocanthion. R = right, L = left.
